# Study of genetic variability, heritability, and genetic advance for yield-related traits in tomato (*Solanum lycopersicon* MILL.)

**DOI:** 10.3389/fgene.2022.1030309

**Published:** 2023-01-04

**Authors:** Adnan Rasheed, Muhammad Ilyas, Taj Naseeb Khan, Athar Mahmood, Usama Riaz, Muhammad Bilal Chattha, Najla Amin T. Al Kashgry, Najat Binothman, Muhammad Umair Hassan, Ziming Wu, Sameer H. Qari

**Affiliations:** ^1^ Key Laboratory of Plant Physiology, Ecology, and Genetic Breeding, Ministry of Education/College of Agronomy, Jiangxi Agricultural University, Nanchang, Jiangxi, China; ^2^ Department of Plant Breeding and Molecular Genetics, Faculty of Agriculture, The University of Poonch, Rawalakot, Azad Jammu and Kashmir, Pakistan; ^3^ Vegetable Crops Research Programme, Horticultural Research Institute, National Agricultural Research Centre (NARC), Islamabad, Pakistan; ^4^ Department of Agronomy, University of Agriculture Faisalabad, Faisalabad, Pakistan; ^5^ Department Entomology, Faculty of Agriculture, The University of Poonch, Rawalakot, Azad Jammu and Kashmir, Pakistan; ^6^ Department of Agronomy, Faculty of Agricultural Sciences, University of the Punjab, Lahore, Pakistan; ^7^ Department of Biology, College of Science, Taif University, Taif, Saudi Arabia; ^8^ Department of Chemistry, College of Sciences and Arts, King Abdulaziz University, Rabigh, Saudi Arabia; ^9^ Research Center on Ecological Sciences, Jiangxi Agricultural University, Nanchang, China; ^10^ Department of Biology, Al-Jumum University College, Umm Al-Qura University, Makkah, Saudi Arabia

**Keywords:** coefficient of variability, genetic advance, heritability, tomato, yield

## Abstract

Tomato is one of the most significant vegetable crops, which provides several important dietary components. Pakistan has a significant low tomato yield compared to other countries because of low genetic diversity and the absence of improved cultivars. The present study aimed to investigate the genetic variability, heritability, and genetic advance for yield and yield-related traits in tomato. For this purpose, eight tomato parents and their 15 crosses or hybrids were evaluated to study the relevant traits. Significant variation was observed for all studied traits. Higher values of the genotypic coefficient of variability (GCV) and phenotypic coefficient of variability (PCV) were recorded for yield per plant (YP) (kg) (37.62% and 37.79%), as well as the number of fruits per cluster (NFRC) (31.52% and 31.71%), number of flowers per cluster (24.63 and 24.67), and single fruit weight (g) (23.49 and 23.53), which indicated that the selection for these traits would be fruitful. Higher heritability (h^2^) estimates were observed for the number of flowers per cluster (NFC) (0.99%), single fruit weight (SFW) (g) (0.99%), and yield per plant (YP) (kg) (0.99%). Single fruit weight (SFW) (g) exhibited higher values for all components of variability. High genetic advance as a % of the mean (GAM) coupled with higher heritability (h^2^) was noted for the yield per plant (YP) (kg) (52.58%) and the number of fruits per cluster (NFRC) (43.91). NFRC and SFW (g) had a highly significant correlation with YP (kg), while FSPC had a significant positive association with YP (kg), and these traits can be selected to enhance YP (kg). Among the 15 hybrids, Nagina × Continental, Pakit × Continental, and Roma × BSX-935 were selected as high-yielding hybrids for further evaluation and analysis. These findings revealed that the best performing hybrids could be used to enhance seed production and to develop high-yielding varieties. The parents could be further tested to develop hybrids suitable for changing climatic conditions. The selection of YP (kg), SFW (g), NFC, and NFRC would be ideal for selecting the best hybrids.

## Introduction

The world population is rising, and there is massive pressure on natural resources (Airoboman and Onobhayedo, 2022). It is now becoming a challenge to feed the growing population (D’Esposito et al., 2021). Tomato is an important vegetable crop, and in its raw form, it is processed to make ketchup and other meals (Kiralan and Ketenoglu, 2022; Kulus, 2022; Zafar et al., 2022). South Mexico is the center of origin of tomato (Campos et al., 2021). Tomato fruit is a significant source of vitamins B1 and B6 and C in the human diet (Mellidou et al., 2021; Rosa-Martínez et al., 2021). During 2020, 186,821 million metric tons of tomatoes were produced on 5,051,983 ha (https://www.fao.org/faostat/en/#data). China, India, the United States of America (USA), and Turkey are the top tomato-producing countries (El-Shafie, 2020). The total tomato production in Pakistan in 2020 was 594,210 tons (http://faostat.fao.org). In Pakistan, the availability of tomato seed for local production is insufficient and requires Pakistan to import large quantities of seed every year to meet its shortfall (Hassan et al., 2021).

Therefore, the evaluation of tomato germplasm is of great importance for crop agronomic and genetic enhancement in the current and future time (Ramzan et al., 2014). The lack of genetic variability and unavailability of high-yielding cultivars are the main reasons for low seed production in Pakistan; hence, it is imperative to increase genetic variability to develop high-yielding tomato cultivars by evaluating available germplasm (Brake et al., 2021; Kulus, 2022). Tomato yield is a multigenic trait and is greatly affected by environmental factors (Wang et al., 2021). The breeders used potential hybridization techniques to obtain tomatoes with high-yield potential.

Genetic diversity is the range of different inherited traits within a species, which is the prerequisite of the breeding program. Genetic diversity leads to the selection of superior cultivars and their traits. Hi (2022) evaluated 24 tomato genotypes to study the genetic diversity for morphological traits using molecular markers, inter-simple sequence repeats (ISSRs), and observed significant variation for studied traits. Genetic variability is well defined as the formation of individuals varying in the genotype. Genetic variability for the tomato fruit was studied in 589 tomato accessions, and this germplasm could be used to develop improved genotypes (Marefatzadeh-Khameneh et al., 2021). These examples showed that genetic diversity is necessary to develop high-yielding genotypes. Genetic diversity between the parental lines increases heterosis, whereas genetic homogeneity between the two parents results in phenotypically uniform F_1_ progeny (Liu et al., 2021).

The components of genetic variability like h^2^ and genetic advance (GA) are essential biometric tools for assessing dissimilarity in population for making a selection (Akhter et al., 2021) and evaluating tomato germplasm for improvement through breeding techniques (Eppakayala et al., 2021). Javed et al. (2022) studied the higher PCV and GCV for yield and yield-related traits in tomato hybrids and indicated the role of genetic variability in plant selection. Understanding the nature of the inherited trait of tomatoes, whether they are phenotypic or genotypic, is essential (Anuradha et al., 2020). Erazo et al. (2020) investigated the higher h^2^ coupled with higher genetic advance for the number of fruits per cluster (NFRC) and single fruit weight (SFW) (g). Other researchers, such as Kumari et al. (2020), observed a moderate behavior of GCV and PCV with higher h^2^ and lowered genetic advance for the number of fruits per cluster (NFRC) and yield per plant (YP) (kg). Maurya et al. (2020) also discussed high genetic variation and h^2^ for days to maturity (DM), the number of branches per plant (NBP), and the number of fruits per plant (NFRP) in tomatoes. Pakistan is facing the issue of low tomato yield because of the lack of sufficient genetic diversity or variability. Therefore, this present study aimed to explore genetic variation, h^2^, and genetic advance for yield and yield-related traits in tomatoes to identify vigorous genotypes that would enhance seed production in Pakistan and lead to self-sufficiency. In this study, several new hybrids were used, which were developed indigenously. These new hybrids could be the potential source for the development of high-yielding tomato cultivars in future research studies. Although the previous study conducted by Ramzan et al. (2014) studied yield-related traits of parents and hybrids, however, in this study, several new hybrids and traits were studied to fulfill the gap which was not previously considered. Fruit setting percentage per cluster is rarely studied in previous studies. This paper provides sound results regarding exploring genetic diversity among tomato genotypes and their hybrids. This can be useful to conduct future studies to identify the high-performing tomato genotypes.

## Materials and methods

The current study was carried out at the experimental field of the Department of Horticultural Research and Development (DHRD), National Agricultural Research Centre (NARC) Islamabad, Pakistan. The experiment was carried out in a randomized complete block design (RCBD). A total of eight parents and their 15 hybrids (Table 1) were chosen for this study. These parents showed significant genetic variation for yield and yield-related traits. The parents were earlier tested in different combinations and selected because of their excellent combining ability to produce hybrids. The top eight best performing parents were chosen, and 15 hybrids were developed. These hybrids were chosen because of their superior performance for all studied traits. Some of these hybrids were tested in an earlier experiment by Ramzan et al. (2014), and not all qualified for the subsequent trials. In the current study, some new hybrids were tested. The current study was conducted to study the genetic diversity, h^2^, and genetic advance for different traits of parents and hybrids.

**TABLE 1:** List of parents and hybrids used in the study.

Parent	Hybrid	Hybrid
17905	Nagina × 17905	Rio Grande × Continental
Rio Grande	Nagina × BSX-935	Pakit × 17905
Pakit	Nagina × Continental	Pakit × BSX-935
BSX-935	Roma × 17905	Pakit × Continental
Roma	Roma × BSX-935	VCT-01 × 17905
VCT-01	Roma × Continental	VCT-01 × BSX-935
Continental	Rio Grande × 17905	VCT-01 × Continental
Nagina	Rio Grande × BSX-935	

### Seed germination and plant shifting

The plot size was 7.5 meter square. The seeds of parents and their hybrids were grown at 30**°**C in a growth chamber for 1 week. After 1 week, the germinated seeds of parents and hybrids were moved to plastic trays, and the trays were kept in the plastic tunnel to maintain the required temperature. Plants were shifted on beds, and the plant-to-plant and row-to-row distance was 50 and 100 cm, respectively. Irrigation of seedlings was carried out regularly to keep the plants fresh and healthy. Weeds were removed to keep the plants in a healthy and safe environment and minimize their effects on plant growth. Several insecticides were used to reduce the risk of insect attacks on tomato plants. No fertilizer was applied to determine the accurate yield capacity of each parent and hybrid. A total of 7 plants were randomly selected from each replication and genotype for data collection.

### Data collection

Random plant selection (seven plants) was made from each replication for each parent and hybrid to record the data for each parameter. Random plants were selected for plant height (PH) from each parent, hybrid, and replication. The ruler was placed at the base of the plant, and PH was measured from the base to the top of the plant. DF (50%) and DM (50%) were recorded by counting the number of flowers and fruits (50% emerged flowers and 50% ripened fruits), respectively, from each replication. To count the NBP, NCP, NFC, and NFRC, plants from each parent, hybrid, and replication were chosen randomly, and the relevant values were recorded. For SFW (g), random plant selection was made from each parent and hybrid from all replications. Fruits were taken from each plant, and the value was recorded using an electronic balance. The data for NLF were noted by counting the number of locules from each fruit of plants selected from parents and hybrids. FSPC was noted by counting the number of fruit set on each chosen plant of the parent and the hybrid. YP (kg) was measured by weighing the fruits of each randomly selected plant of parents and hybrids from each replication.

### Statistical analysis

The analysis of variance (ANOVA) for yield-related parameters was carried out using the procedure proposed by Steel and Torrie (1960). The significance level was checked using 5% and 1% probability. The ANOVA was calculated using MSTAT-C software. The values of parents and hybrids were subjected to ANOVA, and a significance level was observed for all traits. ANOVA showed the level of significance for given traits. Likewise, GCV and PCV indicated a significant amount of variability among the genotypes for all the studied characteristics as calculated using the method of Hallauer et al. (2010). Genetic advance and h^2^ were determined using the method of Hanson et al. (1956). Principal component analysis (PCA) for the major traits was carried out using PAST software to simplify the complexity in high-dimensional data while retaining trends and patterns. Pie charts were also made using PAST software. Pearson coefficient analysis was calculated using IBM SPSS 20.

## Results

### Analysis of variance (ANOVA)

**FIGURE 1:**
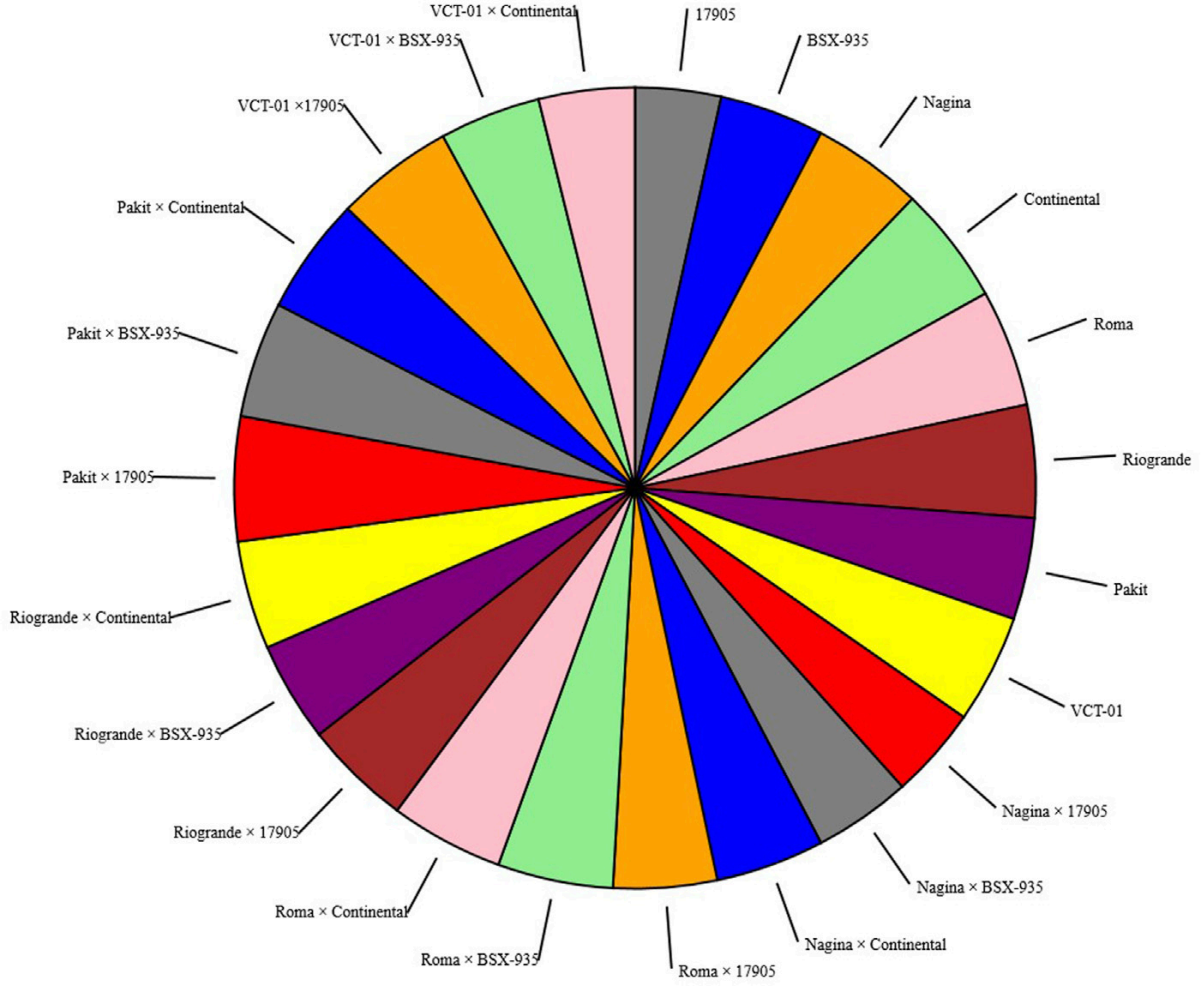
Pie chart represents the size of the value of DM (50%) for each parent and hybrid. Each segment in the pie chart represents a category.

**FIGURE 2:**
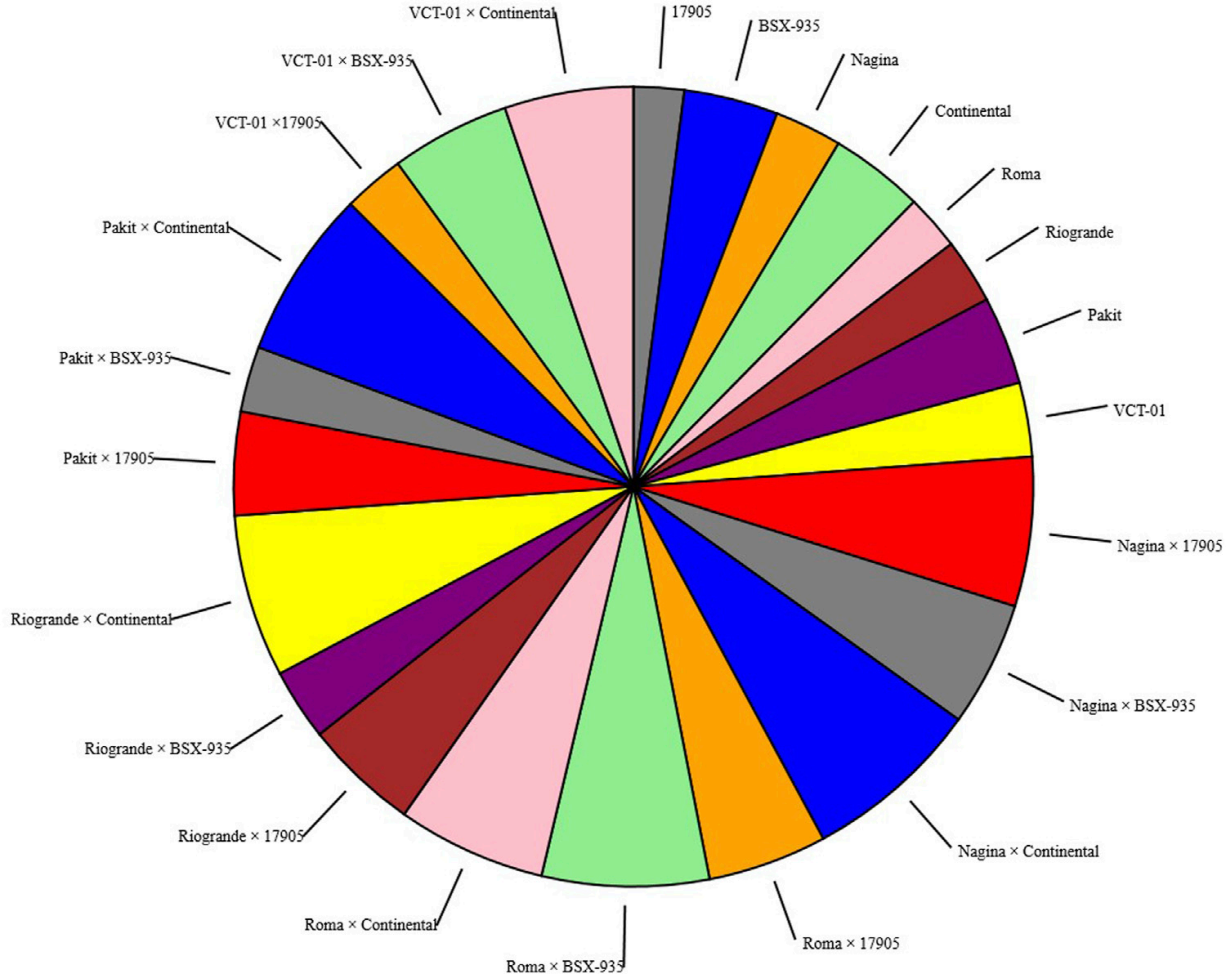
Pie chart shows the different values of a given variable. Pie chart represents the size of the value of YP (kg) for each parent and hybrid.

ANOVA indicated the significant differences among the parents and hybrids for all the studied characters. These differences indicated the existence of variability in germplasm and offered opportunities for the improvement of yield and yield-related traits *via* selection (Table 2). The correlation between yield and yield-related traits is given in Table 3. The mean values for genotypes are presented in Table 4. Pie charts are employed to show fractions of a whole and represent proportions at a set point in time. Pie charts do not show deviations over time. The pie chart for DM (50%) and YP (kg) (Figures 1, 2) of parents and hybrids showed percentages of a whole and represented percentages at a set point in time. The size value of the total amount is divided among distinct categories as a circle (the namesake pie).

**TABLE 2:** Analysis of variance (ANOVA) for different yield traits.

Mean sum of squares				
Trait	Replication	Genotype	Error	Coefficient of variability
PH (cm)	27.1	11083.8**	285.4	3.08%
DF (50%)	0.087	691.072**	28.580	3.20%
NBP	0.0452	18.3814**	0.6281	2.39%
NCP	0.023	203.092**	10.603	2.84%
NFC	0.105	151.912**	1.015	2.47%
NFRC	0.136	155.137**	3.644	5.95%
DM (50%)	1.66	1930.28**	111.97	2.63%
SFW (g)	3.0	17069.8**	114.3	2.36%
NLF	0.0325	16.8681**	1.3075	6.03%
FSPC	28.87	5381.94**	1006.15	6.15%
YP (kg)	0.0318	30.7286**	0.5587	6.24%
	DF = 2	DF = 22	DF = 44	

Note: PH, plant height (cm); DF, days to 50% flowering; NBP, number of branches per plant; NCP, number of clusters per plant; NFC, number of flowers per cluster; NFRC, number of fruits per cluster; DM, days to 50% fruit maturity; SFW, single fruit weight (g); NLF, number of locules per fruit; FSPC, fruit setting percentage per cluster; YP, yield per plant (kg); GCV, genotypic coefficient of variability; PCV, phenotypic coefficient of variability. ** = highly significant; * = significant.

**TABLE 3:** Pearson correlation coefficient among yield and yield-related traits in tomatoes.

Trait	NFRC	SFW	FSPC	YP
NFRC	1	0.758**	0.784**	0.712**
SFW	0.758**	1	0.573**	0.674**
FSPC	0.784**	0.573**	1	0.434*
YP	0.712**	0.674**	0.434*	1

** Correlation is significant at the 0.01 level.

* Correlation is significant at the 0.05 level.

**TABLE 4:** Mean performance of parents and their hybrids for 11 variables.

Parent/hybrid	PH (cm)	DF (50%)	NBP	NCP	NFC	NFRC	DM (50%)	SFW (g)	NLF	FSPC	YP (kg)
17905	64.17	29.33	4.73	17.93	4.06	3.13	48.33	34.07	3.23	77.89	0.846
BSX-935	60.23	23.66	5.30	14.00	5.20	2.20	59.00	57.07	2.50	53.25	1.58
Nagina	63.20	29.33	5.10	15.33	3.36	2.26	62.66	61.07	2.23	68.12	1.13
Continental	68.21	29.00	4.70	16.00	5.00	3.90	66.66	74.10	3.73	78.53	1.58
Roma	66.17	28.00	4.76	18.80	5.50	3.20	66.00	51.40	2.86	58.56	0.930
Rio Grande	71.20	30.00	3.83	17.40	4.00	2.96	63.36	47.20	3.26	74.78	1.09
Pakit	76.13	28.00	4.43	15.20	4.50	3.40	57.33	56.33	2.13	76.12	1.48
VCT-01	78.20	24.00	5.06	17.00	4.70	3.10	61.33	58.50	2.60	65.51	1.24
Nagina × 17905	96.10	19.33	5.80	18.53	7.40	6.30	51.63	72.10	3.50	84.75	2.50
Nagina × BSX-935	93.10	25.00	4.90	15.80	7.30	6.06	54.96	75.97	3.00	82.73	2.10
Nagina × Continental	100.20	20.00	5.53	16.20	7.46	6.40	61.30	83.27	2.00	86.71	3.00
Roma × 17905	93.50	23.66	5.80	18.46	5.80	4.80	58.33	63.17	3.53	82.28	2.00
Roma × BSX-935	92.17	24.00	5.26	18.73	8.16	6.36	65.10	70.27	2.93	78.40	2.80
Roma × Continental	98.43	23.66	5.16	17.33	8.10	6.73	64.00	80.43	2.00	83.47	2.50
Rio Grande × 17905	95.17	24.33	4.40	15.86	6.16	5.00	60.66	66.60	3.30	81.67	1.93
Rio Grande × BSX-935	81.17	26.33	4.30	18.13	7.10	6.10	56.33	74.50	2.60	86.31	1.20
Rio Grande × Continental	88.80	30.33	5.93	18.53	8.00	7.00	61.06	114.30	2.53	87.52	2.73
Pakit × 17905	84.30	26.00	5.43	17.73	7.26	5.90	70.10	61.33	2.90	80.57	1.73
Pakit × BSX-935	73.70	24.66	5.20	15.16	7.00	5.96	65.33	84.13	3.06	88.77	1.10
Pakit × Continental	80.10	25.66	4.63	19.40	6.50	5.36	66.00	83.10	3.46	82.25	2.83
VCT-01 × 17905	86.37	21.00	4.40	15.66	5.20	4.13	66.66	60.10	2.60	79.47	1.03
VCT-01 × BSX-935	90.30	20.33	5.00	21.46	6.60	4.80	57.00	64.10	2.53	72.72	2.00
VCT-01 × Continental	102.27	24.33	5.10	18.80	8.00	6.10	54.10	78.33	3.20	76.57	2.16
LSD value	7.99	2.53	0.37	1.54	0.047	0.090	5.00	5.05	0.54	15.01	0.35

### Principal component analysis (PCA) and Pearson correlation coefficient

**FIGURE 3:**
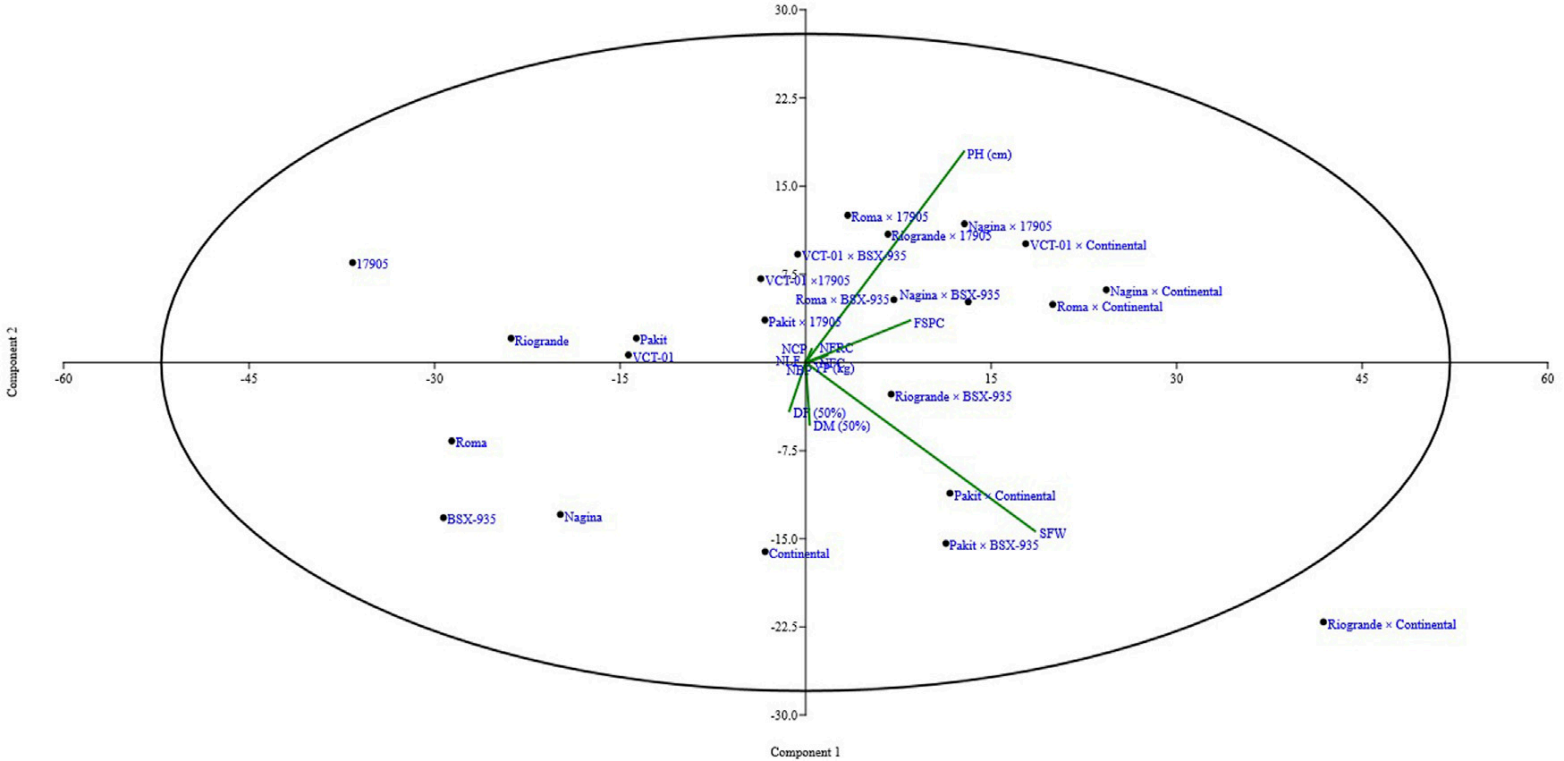
Scatter biplot of different yield and yield-related traits of parents and hybrids. It shows that several variables are correlated with each other and several are not correlated.

**FIGURE 4:**
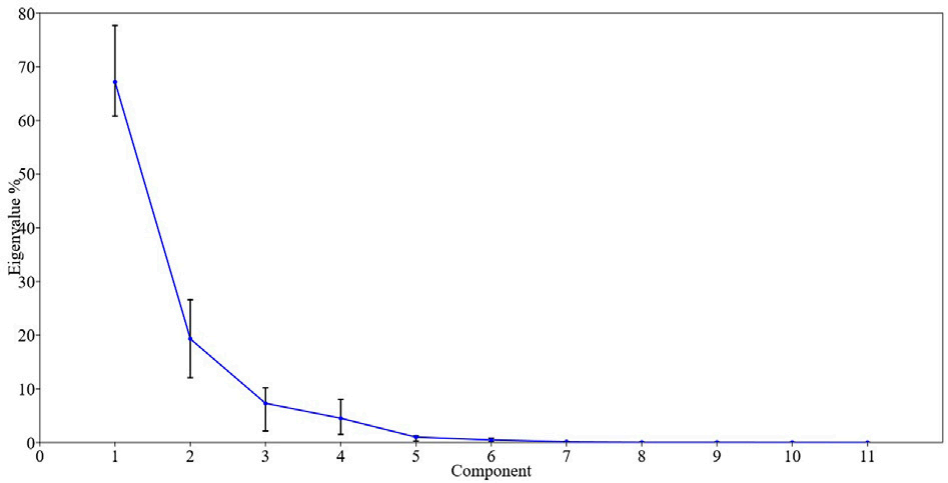
Scree plot for 11 variables of parents and hybrids. It shows that the first four variables could be retained for PCA, while others can be disregarded.

PCA is a statistical analysis used for reducing the dimensionality of such datasets and increasing interpretability while decreasing information loss. Scatter biplot analysis revealed that DM (50%), YP (kg), and SFW (g) are correlated with each other, while PH, FSPC, NFC, and NFRC also had a significant association with each other. DF (50%) had a positive correlation with NBP and a negative correlation with all other variables, while NLF and NCP exhibited a positive association with each other (Figure 3). In scree plot analysis, the eigenvalues are displayed on the *y*-axis and the number of components on the *x*-axis. It shows a downward curve. The point where the slope of the curve is visibly flattening off (the “elbow”) specifies the number of factors the analysis should create. In the current scree plot, PC1, PC2, PC3, and PC4 should be retained in exploratory analysis to keep in PCA, and the rest behind the first three components are disregarded (Figure 4). Correlation represents the inter-relationship between two traits. The correlation of NFRC, SFW, FSPC, and YP showed different ranges from significantly positive to highly significant and positive. In the correlation coefficient analysis, NFRC showed a highly significant positive correlation with SFW (0.758**), FSPC (0.784**), and YP (0.712**). SFW exhibited a highly significant positive association with NFRC (0.758**), FSPC (0.573**), and YP (0.674**). Likewise, FSPC showed a highly significant correlation with NFRC (0.784**) and SFW (0.573**) and a significant positive association with YP (0.434**). A highly significant positive correlation (0.712** and 0.674**) was recorded for YP with NFRC and SFW and a significant positive correlation with FSPC (0.434*) (Table 3). The positive correlation of NFRC, SFW, FSPC, and YP indicated that YP could be improved by directly selecting these traits.

### Mean performance of parents and hybrids

**FIGURE 5:**
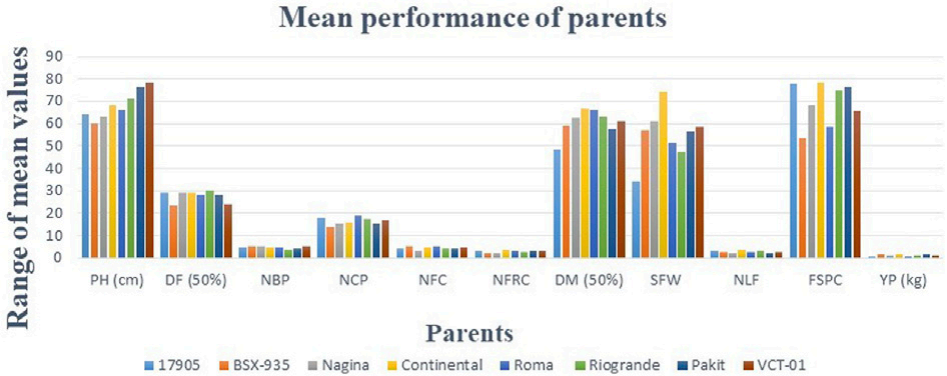
Comparisons of the mean values of parents for 11 variables. A total of eight parents showed different ranges of values for 11 variables. The difference in values of traits among the parents indicated the scope of selection.

**FIGURE 6:**
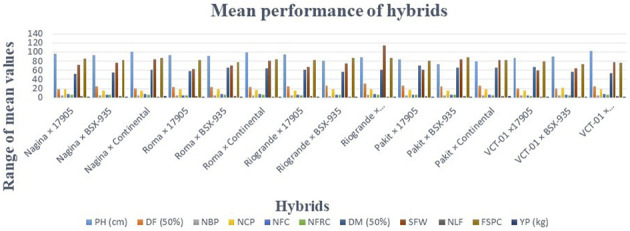
Comparisons of the mean values of hybrids for 11 variables. A total of 15 hybrids showed different ranges of values for 11 variables. The difference in values of traits among the hybrids indicated the scope of selection for varietal development.

The mean values of all parents and hybrids showed different patterns of variation for all traits (Figures 5, 6, Table 4). Among the parents, a maximum value (78.20) of PH was recorded for VCT-01, followed by Pakit (76.13), Rio Grande (71.20), Continental (68.21), Roma (66.17), 17905 (64.17), Nagina (63.12), and BSX-935 (60.23).

Minimum DF (50%) was taken by BSX-935 (23.66) along with VCT-01 (24.00), Pakit (28.00), Roma (28.00), Continental (29.00), Nagina (29.33), and 17905 (29.33). The highest DF (50%) was recorded for Rio Grande (30.00). The lowest DF (50%) is an indicator of the early maturing behavior of parents. BSX-935 had the highest NBP (5.30), along with Nagina (5.10), VCT-01 (5.06), Roma (4.76), and 17905 (4.73). Rio Grande had the lowest NBP (3.83). A maximum NCP was noted for Roma (18.80), 17905 (17.93), Rio Grande (17.40), VCT-01 (17.00), and Continental (16.00). BSX-935 exhibited a minimum NCP (14.00). Regarding NFC, the highest score was witnessed for Roma (5.50), BSX-935 (5.20), Continental (5.00), VCT-01 (4.70), Pakit (4.50), and 17905 (4.06), while Nagina showed a minimum NFC (3.36). Continental had a maximum NFRC (3.90) along with Pakit (3.40), Roma (3.20), 17905 (3.13), VCT-01 (3.10), Rio Grande (2.96), and Nagina (2.26). BSX-935 scored the lowest NFRC (2.20). The lowest NFRC can affect the YP of parents. 17905 had a minimum number of DM (50%) (48.33) along with Pakit (57.33), BSX-935 (59.00), and VCT-01 (61.33). The highest value of DM (50%) was observed for Continental (66.66), which shows the late maturing attitude of this parent. Parents exhibited significant variation regarding SFW, as shown in Table 4. Continental demonstrated the highest SFW (74.10 g) followed by Nagina (61.07 g), VCT-01 (58.50 g), and BSX-935 (57.07 g). The lowest value of SFW was recorded for 17905 (34.07 g). NLF affects the fruit size and shape. More NLF may result in better fruit size. A maximum NLF was witnessed for Continental (3.73), Rio Grande (3.26), and 17905 (3.23). Pakit had the lowest NLF (2.13). FSPC represents the number of fruits that emerged on a cluster. The highest FSPC was exhibited by Continental (78.53), followed by 17905 (77.89), Pakit (76.12), Rio Grande (74.78), Nagina (68.12), VCT-01 (65.51), and Roma (58.56). The parent, BSX-935, had the lowest FSPC (53.25). YP (kg) is the ultimate goal of all breeding programs. Continental and BSX-935 had the highest YP (1.58 kg and 1.58 kg, respectively) along with Pakit (1.48 kg), VCT (1.24 kg), Nagina (1.13 kg), and Rio Grande (1.09 kg). The lowest value of YP among all parents was scored by 17905 (0.84 kg).

Hybrids showed a distinct pattern of performance for the given variables. The maximum value of PH was secured by VCT × Continental (102.27 cm), Nagina × Continental (100.20), Roma × Continental (98.43), and Nagina × 17905 (96.10). The lowest PH was recorded for Pakit × BSX-935 (73.70). Hybrids like Nagina × 17905 and Nagina × Continental showed the minimum number of DF (19.33 and 20.00, respectively). Nagina × 17905 was regarded as an early maturing hybrid as it also exhibited a minimum DM (50%) (51.63). In contrast, Rio Grande × Continental exhibited the highest number of DF (50%) (30.33), which may lead to the late maturing trend of this hybrid. Rio Grande × Continental, Nagina × 17905, and Roma × 17905 scored the highest NBP (5.93, 5.80, and 5.80, respectively), whereas Rio Grande × BSX-935 scored the lowest value of NBP (4.30). VCT-01 × BSX-935 showed the highest NCP (21.46), and the lowest number was attained by Pakit × BSX-935 (15.16). Roma × BSX-935 had the highest NFC (8.16), followed by Roma × Continental (8.10), VCT-01 × Continental (8.00), Rio Grande × Continental (8.00), Nagina × Continental (7.46), Nagina × 17905 (7.40), Nagina × BSX-935 (7.30), Pakit × 17905 (7.26), Rio Grande × BSX-935 (7.10), Pakit × BSX-935 (7.00), and VCT-01 × BSX-935 (6.60). The lowest value was scored by VCT-01 × 17905 (5.20). The maximum number of NFRC was observed for Rio Grande × Continental (7.00), Roma × Continental (6.73), Nagina × Continental (6.40), Roma × BSX-935 (6.36), Nagina × 17905 (6.30), and VCT-01 × Continental (6.10), whereas a minimum number of NFRC was recorded for VCT-01 × 17905 (4.13). All hybrids had different values for DM (50%), where Nagina × 17905 showed an early fruit maturity trend indicated by the lowest value of DM (50%) (51.63) and VCT-01 × 17905 was a late maturing hybrid as revealed by the highest value for DM (50%) (66.66). The highest SFW (g) was observed for Rio Grande × Continental (114.30 g), followed by Pakit × BSX-935 (84.13 g) and Nagina × Continental (83.27 g), and the lowest value was observed for VCT-01 × 17905 (60.10 g). For NLF, the highest value was observed for Roma × 17905 (3.53) and the lowest value for Nagina × Continental (2.00).

Pakit × BSX-935 showed the highest score for FSPC (88.77), followed by Rio Grande × Continental (87.52), Nagina × Continental (86.71), and Rio Grande × BSX-935 (86.31). VCT-01 × BSX-935 showed the lowest FSPC (72.72). Nagina × Continental was a high-yielding hybrid with YP (3.00 kg) along with Pakit × Continental (2.83 kg), Roma × BSX-935 (2.80 kg), Rio Grande × Continental (2.73 kg), Roma × Continental (2.50 kg), and VCT-01 × Continental (2.16 kg). Among all hybrids, the lowest value of YP was observed for VCT-01 × 17905 (1.03 kg).

### Genetic variability, h^2^, and genetic advance

The results of genetic variability indicated that the highest GCV and PCV were observed for YP (37.62% and 37.79%), followed by NFRC (31.52% and 31.71%), NFC (24.63% and 24.67%), and SFW (g) (23.49% and 23.53%), which exhibited the existence of large genetic variability and demonstrated the effective selection for the given traits. The moderate values of GCV and PCV were recorded for NLF (17.34% and 17.68%), followed by PH (15.56% and 15.56%), DF (12.69% and 12.83%), FSPC (11.06% and 11.62%), NBP (10.48% and 10.57%), and NCP (10.01% and 10.15%), respectively. DM had the lowest GCV and PCV (8.72% and 8.90%, respectively), which exhibited a huge impact of the environment on the trait (Table 5).

**TABLE 5:** Genetic variability, heritability, and genetic advance for yield-related traits in tomatoes.

Trait	Mean	GCV (%)	PCV (%)	h^2^ (%)	Genetic advance (%)	Genetic advance as a percentage of mean (%)
PH (cm)	82.74	15.56	15.56	0.98	17.90	21.64
DF (50%)	25.21	12.69	12.83	0.97	4.43	17.59
NBP	4.99	10.48	10.57	0.98	0.72	14.55
NCP	17.28	10.01	10.15	0.97	2.39	13.84
NFC	6.19	24.63	24.67	0.99	2.11	34.19
NFRC	4.83	31.52	31.71	0.98	2.12	43.91
DM (50%)	60.75	8.72	8.90	0.97	7.35	12.10
SFW (g)	68.32	23.49	23.53	0.99	22.23	32.55
NLF	2.86	17.34	17.68	0.96	0.68	23.78
FSPC	77.69	11.06	11.62	0.90	11.46	14.75
YP (kg)	1.80	37.62	37.79	0.99	0.94	52.58

Note: PH, plant height (cm); DF, days to 50% flowering; NBP, number of branches per plant; NCP, number of clusters per plant; NFC, number of flowers per cluster; NFRC, number of fruits per cluster; DM, days to 50% fruit maturity; SFW, single fruit weight (g); NLF, number of locules per fruit; FSPC, fruit setting percentage per cluster; YP, yield per plant (kg); GCV, genotypic coefficient of variability; PCV, phenotypic coefficient of variability; h^**2**^. = heritability.

A high heritability was witnessed for all traits, NFC (0.99%), SFW (0.99%), YP (0.99%), NFRC (0.98%), PH (0.98), NBP (0.98%), DF (0.97), NCP (0.97%), DM (50%) (0.97%), NLF (0.96%), and FSPC (0.90%) (Table 5). The highest value of genetic advance was recorded for SFW (g) (22.23%), which showed the presence of the additive gene action, while moderate GA was detected for PH (17.90%) and FSPC (11.46%) (Table 5), which showed non-additive gene action. NLF had the lowest GA (0.68), followed by NBP (0.72) and YP (kg) (0.94) (Table 5). The results showed that maximum genetic advance as a % of the mean was detected for YP (52.58%), NFRC (43.91%), NFC (34.19%), SFW (g) (32.55%), NLF (23.78%), and PH (21.64%), while DF (50) % (17.59%), FSPC (14.75%), NBP (14.55%), NCP (13.84%), and DM (50%) (12.10%) revealed moderate genetic advance as a % of the mean which stated non-additive gene action.

## Discussion

Tomato is one of the most important vegetables worldwide, presenting a high added value (Azevedo et al., 2022). In the past, many breeders have significantly contributed to the yield by increasing the genetic variability in given tomato populations or cultivars (Rasul et al., 2022). The exploitation of genetic diversity is critical to enhancing tomato production by developing high-yielding cultivars (Ramzan et al., 2014). Therefore, in Pakistan, there is a crucial need for developing improved varieties of tomatoes with high yield and quality features. A substantial yield improvement can be achieved by developing F_1_ hybrids of tomatoes (Aziz et al., 2021).

Genetic advance, h^2^, and genetic variability are used to improve the selection of parents and hybrids. All traits had a higher magnitude of h^2^, which shows that these traits are highly heritable (Table 5). Earlier investigators, such as Eppakayala et al. (2021), studied significant variations for numerous traits in tomatoes. The conclusion in the current study aligned with the earlier research outcomes (Ramzan et al., 2014; Behera et al., 2020). Days to 50% flowering indicated an early maturing attitude of parents/hybrids. Early flowering genotypes lead to early fruit maturity and escape the threats of many abiotic stresses. Genotypes with early flowering and early maturity attributes must have high-yielding potential, and it depends on the combination of strong and vigorous early growth, nutrients, water usage efficiency (WUE), stable photosynthesis and respiration, the production of more biomass before fast anthesis, and effective uptake of metabolites into seeds, all ending in high yield (Passioura, 2012; Shavrukov et al., 2017).

The hybrid, Nagina x 17905, secured the lowest day to 50% flowering (19.33), and this hybrid could be used to develop an early maturing tomato cultivar. The minimum days to fruit maturity were secured by 17905 (48.33), which was regarded as an early maturing parent. In some cases, early maturing cultivars may lead to a high yield and improved quality of tomatoes. These characters are the most significant for the selection criteria. These findings could help future researchers improve tomatoes’ early flowering and fruit-maturing traits to reduce the risk of diseases and shorten the growth duration. The earlier researchers, who worked on some of these hybrids and parents, did not report data on these traits (early flowering and early fruit maturity) (Ramzan et al., 2014). Haydar et al. (2007) studied the days to flowering, and Prajapati et al. (2015) studied the days to 50% fruit set in tomato genotypes. The genotypic and phenotypic coefficients of variability, h^2^, and genetic advance are essential biometric tools used to assess the genetic divergence among the genotypes (Mohamed et al., 2012; Pooja et al., 2022; Sahoo et al., 2022). Genetic variability is the basis for any selection strategy because the larger the genetic variability in the existing population, the greater will be the scope of selection for the improvement of genotypes for the given traits (Mohamed et al., 2012; Hussain et al., 2021).

A higher magnitude of GCV and PCV indicated the scope of selection as more variation results in an effective selection plan (Islam et al., 2022). Usually, the magnitudes of PCV were, to some extent, higher than those of GCV for the given traits, demonstrating the role of the environment in the appearance of the trait. In the current study, YP (kg), NFC, NFRC, and SFW (g) had the highest GCV and PCV. These findings indicated the naturally occurring differences among the parents. They permitted an enormous scope of selection to develop potential cultivars, allow parents/hybrids to adopt environmental changes, and maintain a high-yielding attitude. In the current study, most traits had moderate to higher magnitudes of GCV and PCV. Higher PCV and GCV were previously studied for fruit yield per hectare and average fruit weight (g), which is different from the current findings because the traits studied in both studies are different (Hussain et al., 2021). Haydar et al. (2007) and Mohamed et al. (2012) reported higher values of GCV and PCV for FW (g), Kumari et al. (2007) studied higher genotypic and phenotypic variance for YP (kg), and other studies (Saleem et al., 2013; Singh et al., 2014; Singh et al., 2017) presented related results. Higher GCV and PCV for NFC were observed by Singh et al. (2014). Many vital characteristics showed a higher magnitude of PCV than those of their GCV, showing the more substantial impact of the environment on the appearance of that trait (Kuru Dosegnaw, 2021; Kulus, 2022). Earlier researchers, such as Rani and Anitha (2011), found a moderate magnitude of GCV and PCV for NBP, which strongly supported our findings. The h^2^ determines to what extent a trait is inherited or the degree to which a trait is inherited (Akhter et al., 2021). A high h^2^ indicates that genetics describes a lot of the variation in a trait between different parents and a low heritability, which is nearly zero, specifies that most of the difference is not genetic. A high h^2^ alone is not considered an essential standard for selection, but the likelihood of effective selection increases with high genetic advance (Pooja et al., 2022). An effective breeding program to improve quantitative traits needs reliable h^2^ estimates (Mohamed et al., 2012). YP (kg) (0.99), NFC (0.99%), and SFW (g) (0.99%) recorded higher h^2^ estimates in the current study so that genetic variation can be exploited and these traits can be improved using this selection criterion (Bineau et al., 2021). Venkadeswaran et al. (2020) observed higher h^2^ values for all traits except NBP, which strongly validated our results. Mohamed et al. (2012) also detected higher h^2^ estimates for NFC, which strongly supported the validity of our results.

Genetic advance is another vital biometric tool to decide on selection and shows the scope of selection (Pooja et al., 2022). A higher magnitude of genetic advance and h^2^ is more reliable in forecasting genetic gain under selection (Eppakayala et al., 2021). A higher h^2^ coupled with the medium genetic advance indicated the need for single plant selection to improve the genotypes. The further crossing is obligatory to create desired variations if both components are low in traits (Behera et al., 2020). SFW (g) had higher values for all components of variability. Shankar et al. (2013) also presented the same results for average fruit weight (g). Higher genetic advance with a higher h^2^ for SFW (g) strongly confirmed that additive gene action is present, and the selection of genotypes for the improvement of SFW (g) would be highly effective (Mahebub et al., 2021). The additive gene effect indicated that additive genes play an equal role in the phenotype and genes do not dominate each other. The more the genes are present, the stronger the phenotype will be (Dutta et al., 2013). The selection of superior genotypes would be effective in improving this character. Genetic advance with the moderate behavior coupled with a higher h^2^ was detected for PH and FSPC in the current study, which exhibited the scope of individual plant selection for further improvement. A high h^2^ does not mean higher genetic advances (Rawat et al., 2020). The selection of parents with higher genetic advances and higher h^2^ for yield-related traits is an essential prerequisite (Cholin and Raghavendra, 2021). Most of the traits in the current study had a higher h^2^ with moderate to low genetic advance, which suggests that further selection is required to improve these traits.

A high h^2^ with high genetic advance as a % of the mean was detected for PH, YP, NFC, NFRC, SFW (g), and NLF in the current study, indicating that these parameters could be selected for developing superior genotypes. A higher genetic advance as a % of the mean coupled with high h^2^ is more valuable than h^2^ alone in predicting the resultant effects during the selection of the best genotype (Shankar et al., 2013). Shankar et al. (2013) observed high h^2^ coupled with high genetic advance as a % of the mean for NFRC, NLF, and YP (kg) but low genetic advance for NFRC, YP (kg), and NLF. Likewise, Javed et al. (2022) also observed high h^2^ and GAM for YP (kg), which supported our studies. These traits were highly heritable, which indicated the presence of an additive gene action. A higher h^2^ with low to moderate genetic advance as a % of the mean indicated the effect of the environment on the expression of particular traits (Shankar et al., 2013). These characters could be exploited through heterosis manifestation of dominance and epistatic components. Meena et al. (2018) studied the low and moderate genetic advances as a % of the mean for NBP, DF (50%), and NCP.

High genetic advance and high h^2^ revealed that the environment plays a negligible role in the expression of particular traits as indicated by additive gene action. Hence, these traits can be improved *via* natural selection. Al-Araby (2021) and Soresa et al. (2021) showed dissimilar outcomes for different traits. Biplot and scree plot analyses are critical to understanding genotypes and traits’ similarity and divergence patterns (Rai et al., 2017). Scatter biplot analysis (Figure 3) showed that DM (50) %, SFW (g), and YP (kg) were highly correlated because of their position in the same area, and PC1, PC2, PC3, and PC4, holding the highest eigenvalues, could be regarded for further analysis as depicted by scree plot analysis (Akhter et al., 2021) (Figure 4). Rasul et al. (2022) showed significant variability for fruit weight using PCA and showed that fruit weight selection plays a key role in yield improvement. In the current study, correlation analysis showed that NFRC and SFW (g) had a highly significant positive correlation with YP (kg), and YP (kg) could be improved by direct selection. Direct selection, based on yield components, helps reliability in yield improvement, as mentioned by Kumar et al. (2013). Previous research studies reported the results of components of genetic variability for different yield and yield-related traits. High heritability for all traits makes this study different from previous studies. The use of FSPC is a novel aspect of the current study, and it indicates that YP (kg) can be maximized by increasing this trait. A greater FSPC results in a higher yield. One of the hybrids, Nagina × Continental, had a higher FSPC (86.71%), which also increased YP (kg).

A selection of SFW (g) can enhance YP (kg). A high-yielding hybrid, Nagina × Continental, is one of the core findings of the current study, which showed that this hybrid could be used for varietal development. Further studies are required to enhance the genetic variability to improve the selection program. Hence, the breeder should implement an appropriate breeding procedure to use both additive and non-additive gene effects simultaneously since varietal and hybrid development will go a long way in the breeding programs, especially in the case of tomatoes.

## Conclusion

The main objective of the study was to observe significant variation, heritability, and genetic advance for different traits of parents and hybrids. We have found significant genetic variability for the studied traits. High heritability and genetic advance confirmed that selection could effectively improve traits to increase tomato yield. Current findings confirmed the additive gene action and suggested that the selection of parameters would be effective for further improvement. Nagina × Continental, Pakit × Continental, and Roma × BSX-935 were high-yielding hybrids, while Rio Grande × Continental and Pakit × BSX-935 had higher SFW (g). As shown in the results, superior hybrids indicated that seed production could be maximized to reduce the import of tomato seeds. These hybrids could lead to the development of high-yielding tomato varieties. Likewise, superior parents can be further evaluated to make different cross-combinations. We strongly suggest conducting further studies on these parents and hybrids to validate the results and continue the cultivar development.

## Data Availability

The original contributions presented in the study are included in the article/Supplementary Material. Further inquiries can be directed to the corresponding author.
